# Ovarian cyst torsion in Prader-Willi Syndrome

**DOI:** 10.1186/s12887-023-04223-7

**Published:** 2023-08-08

**Authors:** Ji-cun Zhao, Heng Huang, Hong-lei Gong, Qing-kai Zhao, He Wu

**Affiliations:** 1 Department of General Surgery, Women and Children’s Hospital, Qingdao University, Qingdao, China; 2 Department of Hand and Foot Surgery, The Affiliated Hospital of Qingdao University, Qingdao, China

**Keywords:** Prader-Willi syndrome, Ovarian cyst, Torsion, Case report

## Abstract

**Background:**

Prader-Willi syndrome (PWS) is a genetic disorder involving multiple systems, with an incidence of about 1/10000–25000. Ovarian torsion (OT) is not commonly found in children. Ovarian cyst acts as one of the primary factors resulting in OT. While ovarian cyst torsion with Prader-Willi Syndrome has not been reported before.

**Case presentation:**

A 12-years old female was admitted to Emergency Department of our hospital with the chief complaint of abdominal pain. The outcomes of physical examination revealed the height of 150 cm, weight of 103 kg, BMI of 45.77 kg/m^2^. The patient manifested the special facial features, an obese body, with the abdomen distended into a spherical shape. The fat accumulation in the abdomen significantly embarrassed the palpation. The abdominal CT scan indicated a huge cystic mass in the abdominal cavity, sized about 138 mm × 118 mm. According to medical history, the patient was born with low crying and hypotonia, who has developed the uncontrollable eating behavior since 3-years old. These abnormalities led to a speculation of PWS syndrome, so a genetic test was performed and finally confirmed it, concluding a torsion of ovarian cyst with PWS. With the multidisciplinary consultation, a careful treatment strategy containing the control of blood pressure and blood sugar, coenzyme Q10 was administrated to nourish the myocardium and the application of Growth Hormone was developed. All the above preoperative treatments have brought great benefits to patients. Thus promising the successful completion of operation. The postoperative follow-up till now indicated that the abdominal incision was well healed, without operative complications.

**Conclusions:**

This may be the first case report. In the treatment of ovarian cyst torsion, PWS syndrome requires fully consideration, as the latter can lead to multisystem abnormalities, especially the relation to perioperative management, and even fatalities. Genetic testing should be conducted early when PWS was suspected, accompanied with adequate preparation for the perioperative period, the follow-ups of patients should be maintained for a long time after surgery.

## Introduction

Ovarian torsion (OT) refers to a series of pathophysiology in which the ovary and fallopian tube twist along the vascular pedicle of pelvic infundibular ligament and ovarian proper ligament, which leads to the obstruction of ovarian arteriovenous and lymphatic reflux and perfusion obstruction. The incidence of OT is about 5/100,000 in childhood, which requires emergency surgical intervention in general [1]. Once the diagnosis and treatment are delayed, the ovarian avascular necrosis will inevitably occur [2]. Therefore, a timely diagnosis of ovarian torsion is a significant challenge. Previous studies have demonstrated that the accuracy of diagnosis depending on imaging alone is relatively low, and a combination with clinical symptoms is expected, involving the persistent abdominal pain that lies the most critical manifestation. In addition, it can also occur accompanied by nausea and vomiting, fever, and other discomforts [3]. Ovarian cyst acts as one of the primary factors resulting in OT. Functional ovarian cysts found in children are usually developed as a result of perturbed hormonal stimulation, which often occur during two peak periods: the first year of life and around the point of menarche. And surgery is inevitable if the cyst is so large enough that induces OT [4, 5].

PWS was first proposed by three pediatric doctors in 1956 [6], described as a genetic disorder with multiple system abnormalities referring to gene function defects in the 15q11-q13 region of the paternal chromosome. It is also a typical representative of imprinted inheritance, with the incidence rate about 1/10000–25000 [7]. According to the type of gene defects, it could be divided into mainly the deletion of the 15q11-q13 region of the paternal chromosome, maternal uniparental diploid, imprinting center defect, chromosome translocation, rearrangement [8].

Multiple systems throughout the body are covered by the clinical manifestations of PWS, which are complex and diverse, and lack of specificity, accompanied with the typical age characteristics. Although PWS is poor in the overall prognosis, it leads to a relatively low fatality rate, about 3% [6], generally caused by infection in infants and young children. Therefore, the enrichment of awareness of PWS, punctual diagnose and intervene, can effectively modify its overall treatment effect and prognosis.

Ovarian cyst torsion combined with PWS is rather rarely reported. PWS will lead to the abnormalities of endocrine system, and ovarian cysts are also closely related to hormone secretion. Despite the unclear specific relationship between them, both require high consideration in the treatment. We checked the relevant literature and found that this maybe the first case up to now.

## Case presentation

A 12-years old female was admitted to our hospital with the complaint of abdominal pain. The patient has developed abdominal pain two days before admission, manifesting as intermittent lower abdominal pain, which were aggravated when the body position was changed, without other accompanying discomfort. One day ago, the patient felt the abdominal pain was worsened, so she turned to Emergency Department of our hospital.

By inquiring the patient's medical history, we acquired that she was born with low crying and hypotonia. Unfortunately, this phenomenon did not attract enough attention by doctors and parents at the time, so no examination or treatment was performed on the patient. The patient has also exhibited the delayed development, who learned to sit alone at 1-year old, and started to walk at 2-years old. Since the age of 3-years, she displayed an uncontrollable appetite, accompanied with the significantly increased weight. These abnormal performances have also not attracted sufficient attention from parents. The menarche occurred when she was 11-years old, with a regular period about once every 30 days, and lasting 2–3 day each time. There reported no history of dysmenorrhea, and the time of the last menstrual period could not be accurately described.

The results of physical examination were, body temperature: 38 °C, heart rate: 109 beats/min, breathing: 24 times/min, blood pressure: 130/94 mmHg, height: 150 cm, weight: 103 kg, BMI: 45.77 kg/m^2^, obesity, abnormal facial appearance, disordered behavior, obstructive sleep apnea, soft abdominal muscles, a spherical shape of the distended abdomen. The fat accumulation in the abdomen significantly embarrassed the palpation. Mild tenderness was reported in the right lower abdomen near the pelvic cavity, without rebound pain, abnormal vulvar shape, sparse pubic hair.

**Fig. 1:**
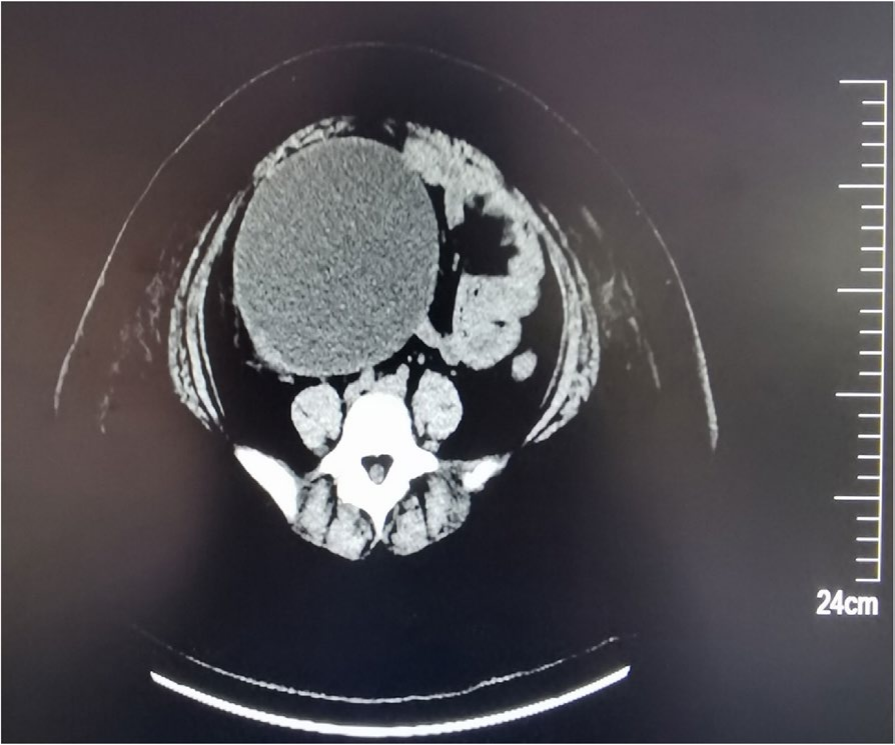
CT-scan: A huge ovarian cyst that occupies the entire abdominal and pelvic cavity, pushing and squeezing the intestinal tube in the abdominal cavity

After admitted to the hospital, the auxiliary examination was performed on the patient, with the result of, Blood-test: WBC18.87 × 10^9^/L, CRP: 224.04 mg/L, Hb1Ac12.9%, BG13.64 mmol/L, Urine-Test: UG 4 + , MAU > 344 mg/L. The results of abdominal ultrasound: a liquid dark area was identified in the right appendage area, about 138 × 118 mm in size, with clear boundary and transparent internal space, the left ovary was normal, without obvious abnormality in the left appendage area. Results of CT-scan: 1. Right lower abdomen to pelvic cavity occupied by cystic space 2. Stone formation in appendicular fecal 3. Intestinal effusion in lower abdomen 4. Multiple small lymph nodes at the root of mesenteric (Fig. 1).

**Fig. 2:**
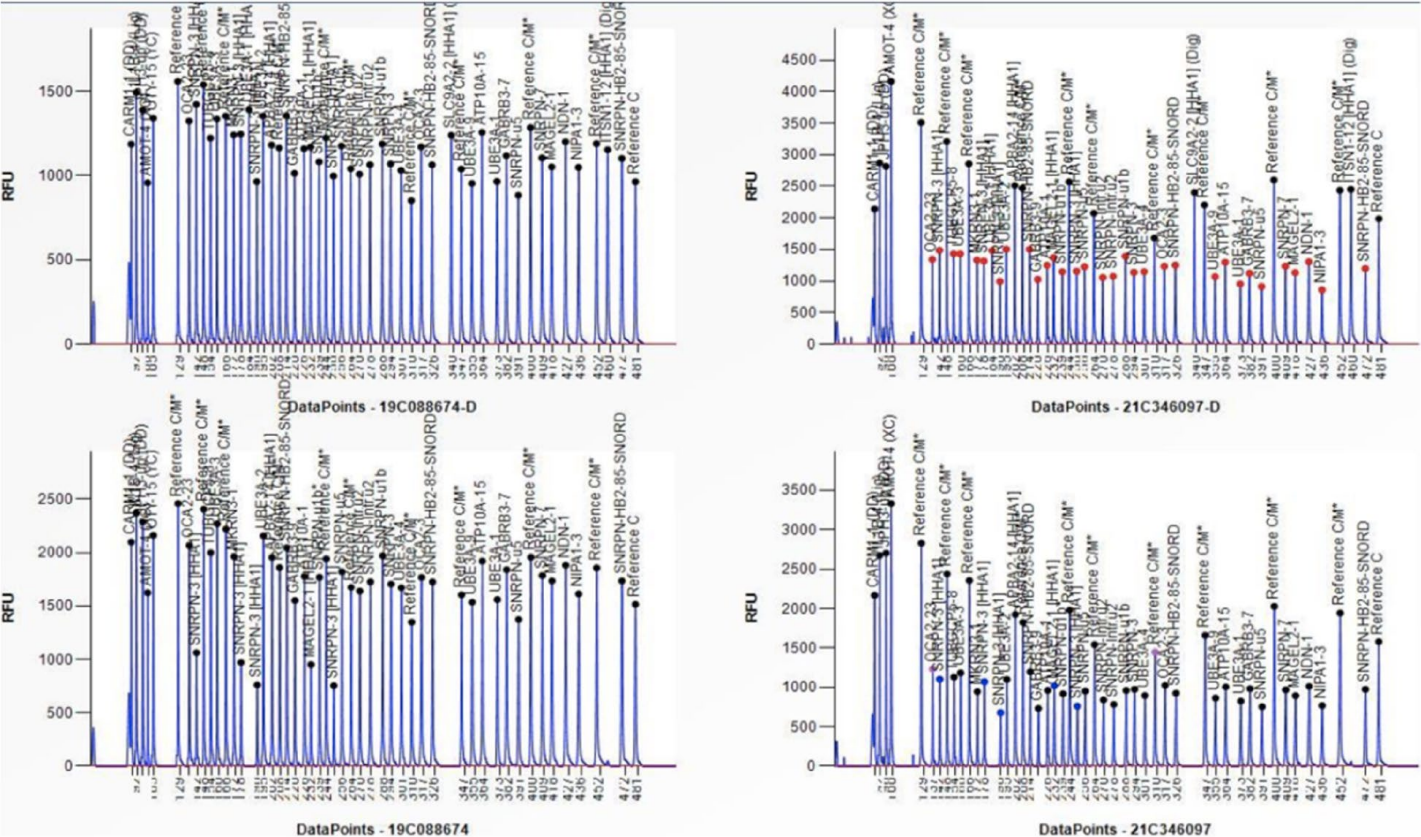
Analytical method: multiplex ligation-dependent probe amplification (MLPA) Analysis result: The results of this test showed that the subject has a gene loss of heterozygosity in the 15q11-13 region, and the methylation test is abnormal. This result supports the subject's pws deletion type

**Fig. 3:**
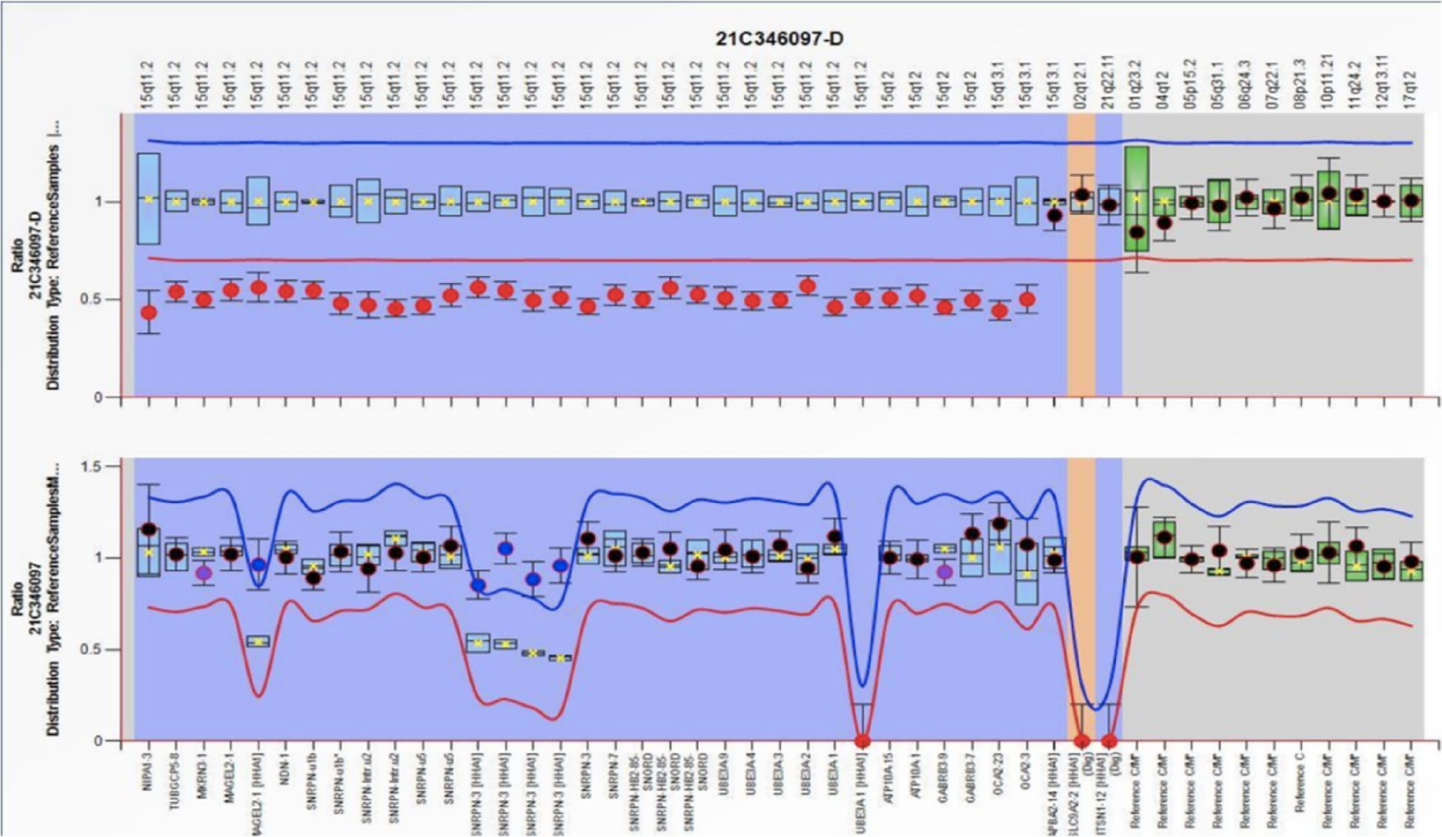
Analysis description: Fluorescence signal intensity between 0.7–1.3 is normal

Considering the unusual history and performance of patient, by combining the clinical manifestations and diagnostic criteria of PWS [6, 7], we speculated an occurrence of PWS. To confirm this conjecture, genetic testing was conducted on the patients. The results of multiple ligation probe amplification (MLPA) detection eventually indicated a loss of heterozygosity in the 15q11-13 region gene and abnormal methylation, which supported paternal deletion of genes at 15q11-q13 (Figs. 2 and 3).

We organized a multidisciplinary consultation covering Anesthesiology, Endocrinology, Respiratory, Cardiovascular unit and Nutrition Section for operation and the following treatment measures were performed: 1. Considering the fever symptoms, abdominal tenderness, and the existence of infection revealed by blood routine examination showed, Cefazolin sodium injection anti-infective treatment was provided. 2. The patient exhibited high blood pressure and a kidney damage showed by urine tests, for which benazepril is administrated to control blood pressure. 3. Metformin was given to intervene the hyperglycemia of patient. 4. The patient was overweight with the increased circulatory load, so coenzyme Q10 was administrated to nourish the myocardium. 5. The application of Growth Hormone has been proved to produce many benefits for PWS, which was also provided for therapy. 6. Diet and exercise instructions were specially formulated for patients to facilitate better weight control.

**Fig. 4:**
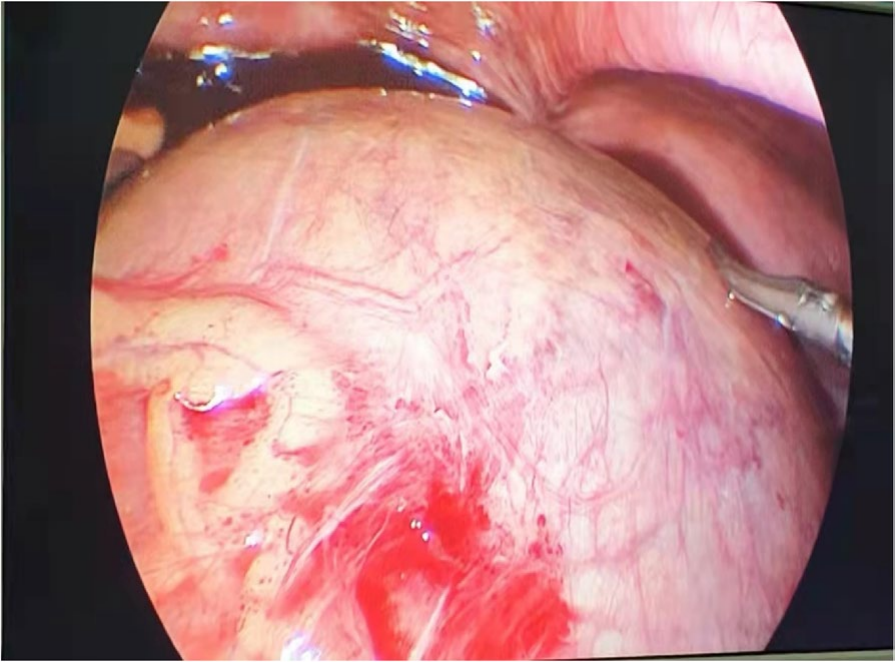
Ovarian cyst is huge, occupying the entire abdominal cavity, close to the liver

**Fig. 5:**
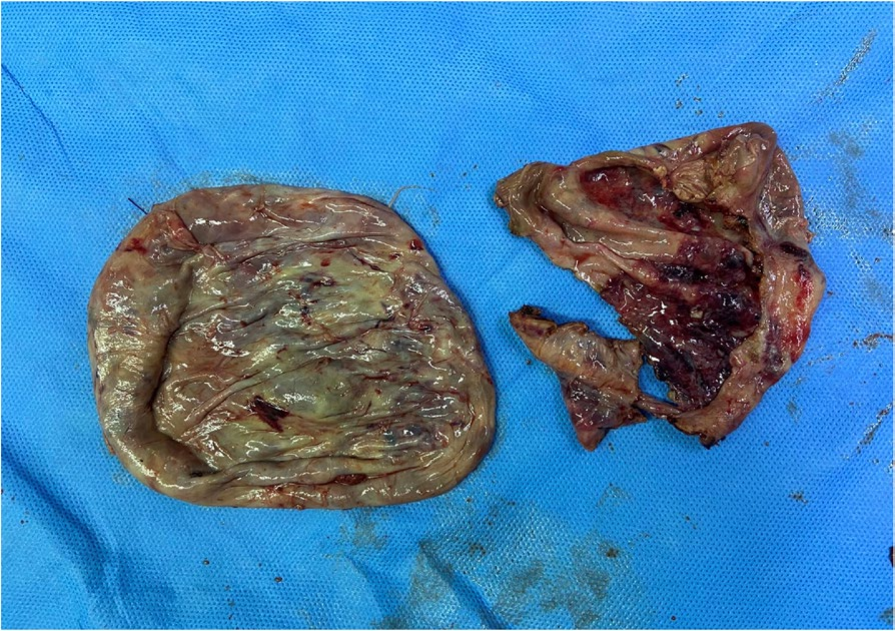
Complete removal of ovarian cysts

**Fig. 6:**
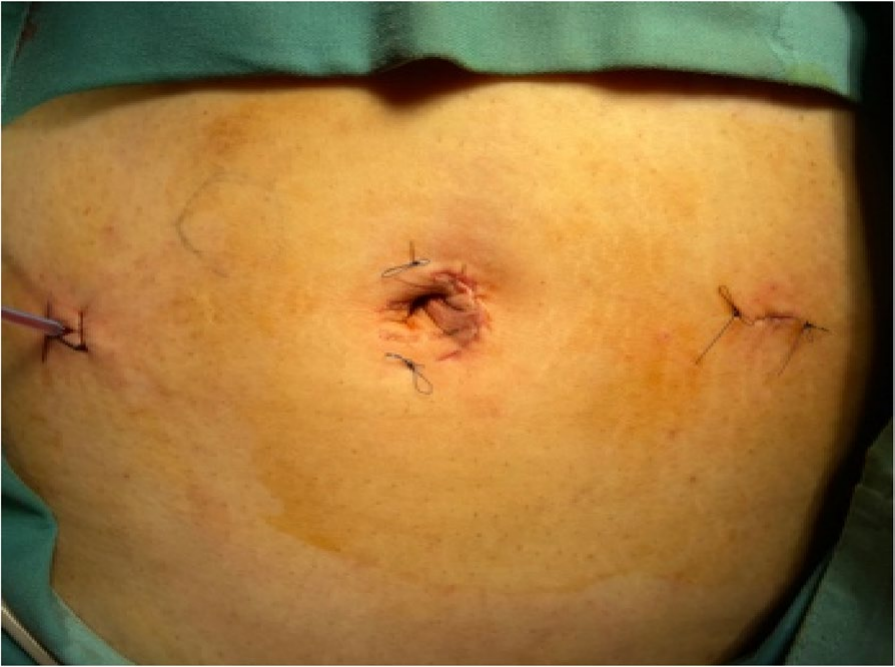
Small surgical incisions, with drainage tube from the right incision

Finally, the operation was performed under the general anesthesia. During the operation, 10 mm Trocar was inserted through the para-umbilical approach, with 5 mm Trocar placed on the left and right sides of the umbilicus. The exploration on right ovary was conducted, revealing a cyst about 20 cm × 15 cm × 10 cm, together with the fallopian tube, rotate 180 degrees counterclockwise. The cyst fluid during the operation was evacuated to be about 1500 ml, the torsion fallopian tube was reset, the cyst was completely peeled off from the ovary, the ovarian cortex was trimmed, followed by the rebuilt ovarian shape, the pelvic drainage tube was placed passing through the umbilical cord. The tissue was loaded into a specimen bag, the specimen was cut into small pieces at the incision and removed, then the incision was sutured. The lesion was finally removed from the side incision and the incision was sutured. The blood lost during the whole procedure was approximately 15 ml. Postoperative pathology: Ovarian cyst, cystadenoma ovarii serosum (Figs. 4, 5 and 6).

After the operation, the patient was admitted to the Pediatric Intensive Care Unit (PICU) for respiratory management, the tracheal intubation was withdrawn at that night and the patient returned to the general ward the next day. Eating was started on the first day after the operation and PWS treatment was continued accompanied with Guidance of diet and exercise, control of blood pressure and blood sugar. Coenzyme Q10 was continued to nourish myocardium and Growth Hormone was used regularly.

**Fig. 7:**
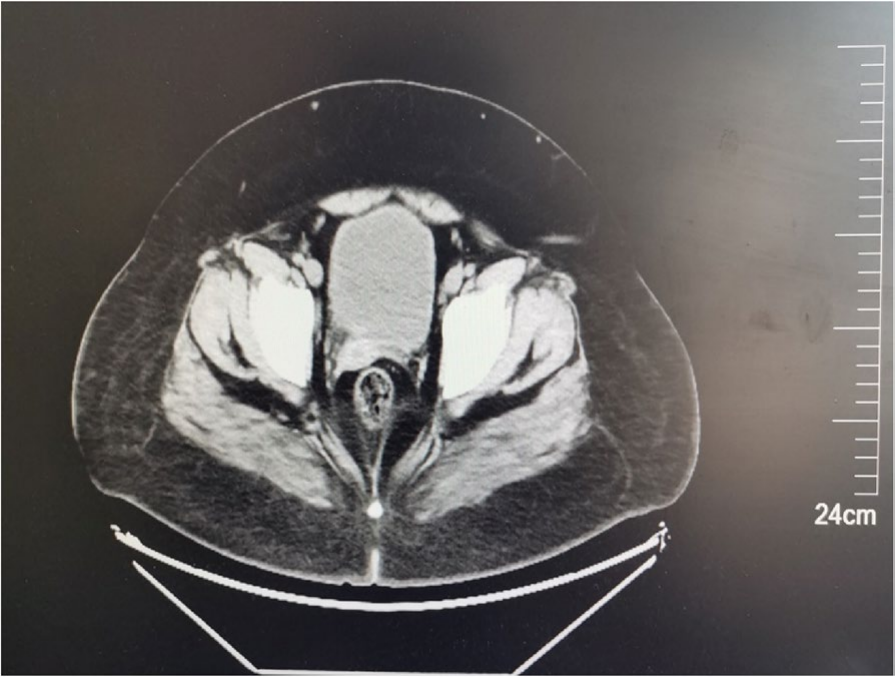
CT-scan (follow-up): The ovarian cyst disappeared

After 7 days, the patient was cured and discharged, followed by the postoperative follow-up till now. According to the present outcomes, the patient has a good recovery, which is mainly revealed by two aspects. First, the surgical recovery is considerable, with the beautiful abdominal scar, without signs of recurrence indicated by the abdominal CT, or occurrence of surgical complications. Second, for PWS, guidance endocrinology and nutrition is continued to be received by the patient, the weight control is currently satisfactory, the BMI index dropped significantly to 38.67 kg/m^2^, accompanied with the satisfactory blood pressure and blood sugar control, and outpatient review is regularly carried out (Fig. 7).

## Discussion and conclusion

The clinical manifestations of PWS are complex, also differing in region and ethnicity. Therefore, the diagnostic level also varies among different countries and regions [9]. At present, the average diagnosis age of PWS reported abroad ranges from 18 days to 4.9 years old [10, 11], and 14 days to 14 years old according to the domestic reports [9].

Especially in remote and poor areas, most patients will not be diagnosed and treated until obesity occurs, while the relatively late treatment results in a low overall quality of life. The occurrence of this situation is generally resulted by the lack of understanding of clinicians in various regions or the improper selection of genetic testing methods. Therefore, the enrichment of awareness of PWS and punctual diagnose and intervene can effectively modify its overall treatment effect and prognosis. The case reported here is a typical example of a lag in diagnosis due to the insufficient awareness. In fact, in the appearance of symptoms with hypotonia and feeding difficulties in the neonatal period, the existence of PWS should be considered by clinicians.

Genetic testing required for PWS diagnose includes [12]: chromosome karyotype analysis, fluorescence in situ hybridization (FISH), methylation-specific polymerase chain reaction MS-PCR, and methylation-specific multiplex probe amplification technology MS-MLPA, etc.

In terms of the treatment of PWS, considering the involvement of the abnormal performance of multiple systems throughout the body, a comprehensive management model covering multidisciplinary participation is recommended, mainly referring to the treatment of metabolic abnormalities, such as dietary behavior intervention and nutritional management, sex hormone replacement therapy, growth hormone and other endocrine problems [13]. Some scholars have pointed out that if the obesity in PWS patients could be effectively controlled, their life span can nearly reach to normal. However, there is currently no such drug to achieve this. It is worth mentioning that stomach volume reduction surgery still remains controversial and is not recommended [14].

Therefore, a strict food manage is recommended with the participation of doctors and parents, so as to ensure a regular diet and a reasonable nutritional structure. At the same time, an appropriate exercise plan should also be formulated to help patients control weight efficiently.

Surgery is currently adopted as the gold standard for the diagnosis of ovarian torsion, which can achieve to determine the degree of torsion and determine tissue activity, in which the timing of surgery is rather critical [2].

Auxiliary examination can further contribute to the diagnosis of mass. However, due to the low specificity of clinical manifestations, an accurate diagnosis is still difficult to achieve. Ovarian cysts act as one of the essential factors leading to ovarian torsion. Prepubertal hormone secretion disorders and Ovulation dysfunction during puberty can induce the occurrence of ovarian cysts. According to the current literature, the relation between menstrual cramps and ovarian torsion still remains uncertain.

Most scholars hold that ovarian torsion is more commonly found after menstruation, accounting for 84% of the total, believing that it is associated to the increased incidence of ovarian cysts after menstruation [15, 16].

In addition, the diameter of ovarian masses is also associated to OT. As the literature reports, OT should be alerted for people with a diameter of ≥ 5.5 cm [17]. The case we reported is basically consistent to those reported in literature.

In summary, it may be the first case report of PWS combined with ovarian cyst torsion. The diagnosis of PWS was so delayed for the patient and the opportunity for early intervention and treatment were missed. Therefore, we hope that the case here will highlight the importance of early diagnosis of PWS. Obesity and endocrine abnormalities are considered one of the common causes of ovarian cysts, as well as the common clinical symptoms of PWS patients. During the treatment of ovarian cyst torsion, the characteristics of PWS should be taken into consideration. Genetic testing should be carried out punctually, the advantages of multidisciplinary consultation should be fully utilized, as well as adequate preparation for the perioperative period, the concept of rapid recovery medicine should be adhered to, accompanied with a minimally invasive surgery, so as to avoid the occurrence of unplanned secondary operations as far as possible. As ovarian cysts still have a certain probability of recurrence, and the treatment of PWS is a long-term process, the follow-up of patients requires to be lasted for a long time after surgery.

## Data Availability

All data generated or analysed during this study are included in this published article and its supplementary information files.

## Abbreviations

PWS: Prader-Willi syndrome
OT: Ovarian torsion
MLPA: Multiple ligation probe amplification
BMI: Body Mass Index
WBC: White blood cell
CRP: C reactive protein
Hb1Ac: Glycosylated Hemoglobin
BG: Blood glucose
UG: Urinary Glucose
MAU: Micro-albumin Urine
CT: Computed tomography
FISH: Fluorescence in situ hybridization
PICU: Pediatric Intensive Care Unit
MS-PCR: Methylation specific polymerase chain reaction
MS-MLPA: Methylation specific multiplex ligation-dependent probe amplification
